# Systematic Evaluation of Vision Transformers for Automated Cervical Cancer Classification: Optimization, Statistical Validation, and Clinical Interpretability

**DOI:** 10.3390/cancers18132178

**Published:** 2026-07-07

**Authors:** Nisreen Albzour, Sarah S. Lam

**Affiliations:** School of Systems Science and Industrial Engineering, Binghamton University, Binghamton, NY 13902, USA

**Keywords:** vision transformer, cervical cancer, pap smear, interpretability, Grad-CAM, medical image classification

## Abstract

**Background/Objectives:** Manual Pap smear analysis for cervical cancer screening is limited by inter-observer variability, time constraints, and restricted expert availability. Although convolutional neural networks (CNNs) have automated cervical cell classification, they remain limited in modeling long-range spatial dependencies and often lack clinical interpretability. **Methods:** In this study, Vision Transformer (ViT) architectures were systematically optimized to enhance automated cervical cancer screening and improve interpretability. The Herlev dataset (917 images: 242 normal, 675 abnormal) was utilized to optimize ViT-Tiny, a lightweight ViT architecture designed for reduced computational complexity, through a comprehensive evaluation of augmentation strategies, class weighting, and hyperparameters. **Results:** The optimal configuration achieved a cross-validation accuracy of approximately 95% (94.89% for the best replicated configuration), in which random horizontal flipping and class weighting (0.7 × 1.3) were identified as most effective. Gradient-weighted Class Activation Mapping (Grad-CAM) analysis confirmed that model attention corresponded to clinically relevant morphological features, including nuclei regions, cell boundaries, and chromatin texture, which align with cytopathological criteria. **Conclusions:** These findings indicate that Vision Transformers can deliver accurate and interpretable decision support for cervical cancer screening by combining competitive classification performance with attention-based transparency relevant to medical AI. Further validation on larger, multi-center datasets remains necessary before clinical deployment.

## Simple Summary

Cervical cancer screening can save lives by detecting abnormal cells early, but manual Pap smear examination is time-consuming and varies among reviewers. This study evaluated a lightweight Vision Transformer model for classifying Pap smear cell images as normal or abnormal. The best-performing model achieved high accuracy and produced visual explanations showing the image regions that influenced its predictions. These explanations often highlighted clinically relevant features, including nuclei and cell boundaries. The findings suggest that lightweight Vision Transformer models may support automated cervical cancer screening, although validation on larger and more diverse clinical datasets is still needed before real-world use.

## 1. Introduction

Cervical cancer remains a significant global health challenge, in which over 600,000 new cases and 340,000 deaths are reported annually worldwide [1]. The primary strategy for reducing mortality is early detection through Pap smear screening, which enables identification of precancerous lesions before they progress to invasive cancer. However, manual analysis of Pap smear slides is time-consuming, labor-intensive, and subject to substantial inter-observer variability, in which diagnostic accuracy varies significantly among cytotechnologists [2,3]. These limitations highlight the critical need for automated, reliable, and interpretable diagnostic solutions that can augment human expertise while maintaining the accuracy essential for clinical decision-making.

Deep learning approaches, particularly convolutional neural networks (CNNs), have revolutionized medical image analysis by providing automated feature extraction and achieving remarkable diagnostic accuracy across various medical imaging modalities [4,5]. CNNs have demonstrated strong performance in cervical cancer detection because they learn hierarchical feature representations directly from image data, which enables them to outperform traditional machine learning methods [6,7]. However, CNN architectures also have inherent limitations in medical imaging applications. Their reliance on local receptive fields and hierarchical feature extraction can fail to capture important long-range spatial relationships that may be crucial for accurate diagnosis of cellular abnormalities [8,9]. Additionally, the limited interpretability of CNN decision-making processes poses significant barriers to clinical adoption, where understanding the rationale behind diagnostic predictions is essential for building trust and enabling clinical validation [10,11].

Related AI-driven automation-oriented work in healthcare has explored hyperautomation-based approaches for leukemia detection and classification [12], which highlight the broader applicability of AI-driven clinical decision-support workflows across diverse disease domains. In a closely related study that applied rigorous evaluation protocols to structured clinical data, ref. [13] evaluated hybrid deep learning–gradient boosting ensembles for breast cancer survival prediction using SEER and METABRIC cohorts. Using nested cross-validation and Nadeau–Bengio corrected statistical testing, the authors found that the hybrid ensembles did not outperform a class-weighted logistic regression baseline. They also observed that performance degradation across cohorts was primarily driven by calibration rather than discrimination. These findings demonstrate that rigorous evaluation methodologies are as important as model architecture selection in AI-driven clinical applications.

A complementary study using the same cohorts further demonstrated that, under cross-cohort prevalence shifts, calibration and feature-level interpretability exhibit distinct behaviors [14]. Specifically, probability calibration was found to be cohort-specific and required local recalibration, whereas SHAP-based feature-importance rankings remained relatively stable across cohorts. These results suggest that predictive performance and explanation quality should be evaluated separately in clinical AI systems. The same principle motivates the interpretability-focused evaluation framework developed in this study for automated cervical cancer screening.

Unlike CNNs, Vision Transformers (ViTs) have emerged as a promising alternative architecture that addresses many limitations of traditional CNNs. By adapting the transformer architecture from natural language processing to computer vision, ViTs [15] leverage self-attention mechanisms to capture global contextual relationships across entire images, which enable more comprehensive analysis of spatial patterns [16]. This global perspective is particularly valuable for medical image analysis, where diagnostic features may be distributed across different regions of an image and require integration of multiple cellular characteristics [17]. The attention mechanisms inherent in ViTs provide natural interpretability through attention maps, which offer insights into which image regions influence diagnostic decisions—a crucial requirement for clinical acceptance and regulatory approval [18]. Recent studies have shown that Vision Transformers can capture both global and local context information while maintaining strong predictive performance, computational efficiency, and robustness when trained on limited labeled datasets in medical imaging applications [19,20].

Despite the promising potential of ViTs in medical imaging, their application in cervical cancer screening remains underexplored. Existing studies have not comprehensively examined optimization requirements for ViTs in this domain, nor have they provided the rigorous statistical validation necessary for clinical adoption. Critical gaps remain in the evaluation of data augmentation strategies, class imbalance handling techniques, and hyperparameter optimization tailored for cervical cell classification. Furthermore, the interpretability advantages of ViTs have not been fully investigated in the context of cytopathological analysis, where alignment with established diagnostic criteria is essential.

This study addresses these gaps by presenting a systematic and clinically oriented framework for evaluating ViT-based models for automated cervical cancer screening using Pap smear images. The novelty of this work lies not in proposing a new transformer architecture, but in developing a reproducible evaluation pipeline that integrates clinically relevant task formulation, model optimization, statistical validation, and interpretability analysis. Specifically, this study makes four primary contributions: (1) reformulating the Herlev cervical cytology dataset into a clinically meaningful binary screening task that distinguishes normal from abnormal cells; (2) systematically evaluating the effects of augmentation strategies, class-weight configurations, and hyperparameter settings on ViT performance using stratified cross-validation; (3) applying statistical tests to determine whether differences among the top-performing configurations are statistically significant; and (4) incorporating Grad-CAM-based interpretability analyses to examine both correctly classified and misclassified cases in relation to cytopathological diagnostic features. Collectively, these components provide a transparent and reproducible framework for assessing the reliability and interpretability of ViT models for cervical cancer screening.

The remainder of this paper is organized as follows: Section 2 reviews prior work on traditional machine learning, deep learning, and emerging transformer-based methods for cervical cancer detection. Section 3 provides a detailed description of the methodology, which includes dataset preparation, model architecture, optimization strategies, and interpretability framework. Section 4 presents the experimental results and discussion, which include statistical analysis and interpretability findings. Section 5 summarizes the key findings, limitations, and directions for future work.

## 2. Related Literature

**Figure 1:**
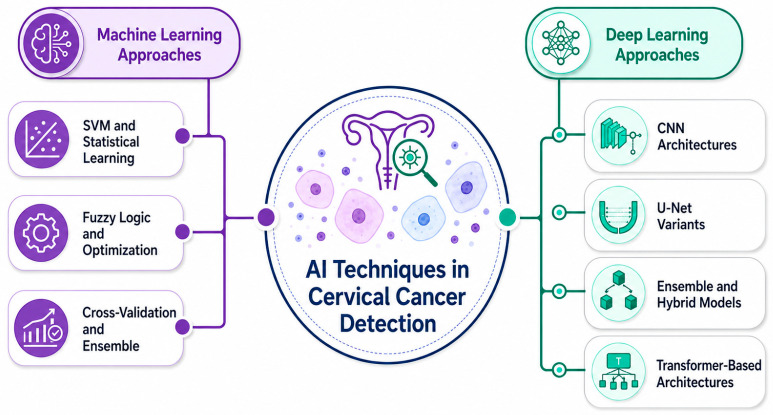
AI techniques in cervical cancer detection.

The application of artificial intelligence techniques in cervical cancer detection and classification has advanced significantly over the past decade, which led to the development of increasingly accurate and automated diagnostic methods. As illustrated in Figure 1, these techniques can be broadly categorized into machine learning and deep learning approaches, in which each category encompasses a range of methodologies tailored to improve diagnostic performance.

### 2.1. Machine Learning Approaches

Traditional machine learning approaches for cervical cancer detection rely heavily on handcrafted feature extraction followed by classical classification algorithms. This subsection reviews studies that employ feature-engineering and machine learning techniques for cervical cytology classification. Support Vector Machine (SVM) and statistical learning approaches have been extensively utilized. Several studies [21,22] developed hybrid linear iterative clustering with Bayes classification, k-Nearest Neighbors (k-NN) with fuzzy logic, and machine learning-assisted detection frameworks, respectively. Mariarputham and Stephen [23] used handcrafted texture features with SVM and neural networks on the Herlev dataset, which found that SVM performed best. Optimization-based methods [24] explored quantum hybrid Particle Swarm Optimization (PSO) with fuzzy k-NN for feature selection and cell classification. Cross-validation and prediction-based machine learning techniques [25,26] investigated stratified k-fold cross-validation, early detection frameworks, and prediction using various machine learning methods. Additional ML approaches addressed Pap smear image classification and early prediction [27,28]. Nandanwar and Dhonde [29] proposed a hybrid stacked ensemble model that combines feature extraction with segmentation, followed by classifier-level fusion using Histogram and Hu Moments techniques.

Traditional machine learning approaches in cervical cancer diagnosis often lack integrated data augmentation strategies, which limits their ability to generalize across diverse image variations. In addition, these systems may be prone to higher false-negative rates, which can result in missed diagnoses and pose risks in clinical settings. These limitations highlight the need for more robust, automated, and generalizable approaches for cervical cancer screening.

### 2.2. Deep Learning Approaches

Deep learning approaches have demonstrated strong performance in medical image analysis, particularly for cervical cancer detection and classification. This subsection reviews studies that employ various deep neural network architectures for cervical cytology classification.

CNNs are widely used deep learning models because of their ability to extract spatial and hierarchical features from image data, which makes them suitable for pattern recognition tasks. CNN architectures have been extensively applied to cervical cancer detection across multiple imaging modalities. Several studies [30,31] employed various CNN variants that included Mask Region-based Convolutional Neural Networks (R-CNN) with Visual Geometry Group (VGG) and Residual Network (ResNet) components, Capsule Networks (CapsNet) preprocessing with VGG-like networks, specialized CNN architectures using deformable Faster Region-based Convolutional Neural Network with Feature Pyramid Networks (R-CNN-FPN) with pretrained backbones for cervical image analysis, and improved Inception-ResNet-V2. Transfer learning approaches [32] implemented and optimized Squeeze-and-Excitation Residual Network-152 (SE-ResNet152) models for multi-class cervical cancer detection. Recent CNN-based work [33,34] explored privacy-preserved detection, lightweight CNN architectures, ResNet-based automated screening, and Attention-Fused Squeeze-and-Excitation Network (AF-SENet) using pretrained models for feature fusion. Additional CNN approaches [35,36] investigated various CNN variants with different backbone networks for enhanced performance.

In a closely related cervical cytology study, Albzour and Lam investigated deep-learning-based segmentation and classification of Pap smear images for cervical cancer detection [37], which provides a relevant foundation for the present transformer-based extension.

U-Net is a deep learning architecture originally developed for biomedical applications, which is recognized for its encoder–decoder structure and ability to capture both high-level context and fine-grained details. U-Net variants and advanced architectures have shown exceptional performance in cervical cancer applications. Enhanced U-Net architectures [38,39] incorporated residual SE blocks, feature fusion from multiple pretrained models, and encoder-weighted designs for improved analysis.

Ensemble learning is a machine learning technique that combines predictions from multiple models to achieve better performance and generalization than any individual model alone. In cervical cancer classification, ensemble and hybrid deep learning approaches have emerged to improve diagnostic accuracy and model robustness. Several studies have proposed hybrid frameworks that integrate deep feature fusion, pretrained neural networks, and classifier-level ensembles to enhance classification performance [40,41]. In addition, radiomics-guided deep learning approaches have combined handcrafted feature descriptors with learned neural network representations to further improve feature expressiveness and diagnostic robustness [42].

Building on advances in CNNs, U-Nets, and ensemble learning, transformer-based architectures have recently gained significant attention because of their capability to capture global contextual relationships [15,43]. Recent studies have further advanced ViT frameworks through systematic optimization strategies and attention-enhanced architectures to improve classification accuracy, computational efficiency, and contextual feature learning in medical image analysis [44,45].

ViTs have demonstrated strong potential for modeling long-range dependencies in medical images, although their performance often benefits from large-scale pretraining and access to substantial training datasets. In a companion benchmarking study, the present authors compared a compact Vision Transformer (ViT-Tiny) with four widely used CNN baselines on the Herlev dataset using a uniform training recipe. The results showed that the ViT-Tiny achieved accuracy comparable to that of the best-performing CNN models while offering substantial advantages in parameter efficiency and memory usage [46].

Recent transformer-based and deep-learning studies further illustrate the range of approaches in this domain. Darwish et al. [47] enhanced a Vision Transformer with shifted patch tokenization on the MobileODT colposcopy dataset, which reports 91% accuracy on a single 70/30 split. Sharma et al. [48] used pretrained CNN feature extraction with VGG19 and ResNet50 combined with a Naive Bayes classifier, reporting 91.15% testing accuracy on the Herlev dataset. Himabindu et al. [49] combined a Swin Transformer with a deep-learning ensemble and autoencoder-based optimization for colposcopy screening. Al-Heiri et al. [50] introduced a hybrid Vision Transformer with an ensemble of DenseNet201, Xception, and Inception-ResNetV2 on the Mendeley LBC and SIPaKMeD datasets across nine cell categories. In contrast to these approaches, which emphasize architectural ensembling, specialized tokenization, or feature-extraction pipelines with classical classifiers and are often validated using dataset-specific train/test protocols, the present study isolates the effect of systematic augmentation, class-weighting, and hyperparameter optimization on a single compact transformer evaluated under stratified cross-validation with replication, statistical testing, and Grad-CAM interpretability analysis.

Despite their successes, deep learning approaches still face several limitations that may impact their clinical applicability. Common challenges include class imbalance between normal and abnormal cells, and limited interpretability through saliency maps or attention visualizations. These issues can reduce model robustness and make predictions less transparent and more difficult to validate for clinical use. This study specifically addresses two critical limitations: class imbalance through systematic optimization of class weighting approaches and interpretability through Grad-CAM visualization aligned with established cytopathological criteria. Addressing these limitations is essential for improving trust, generalizability, and the practical deployment of AI models in cervical cancer diagnosis.

## 3. Methodology

**Figure 2:**
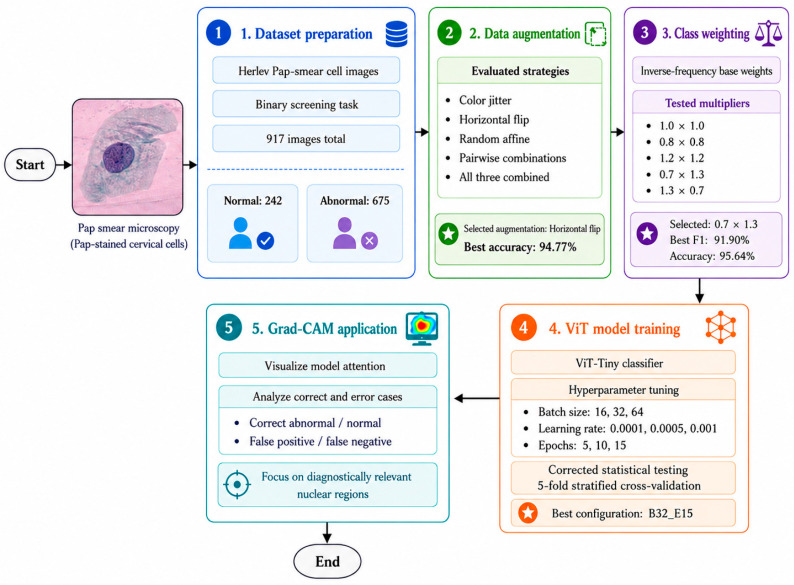
Overview of methodology.

Figure 2 provides a comprehensive overview of the entire methodology employed in this study. Each stage plays a crucial role in ensuring the effectiveness and robustness of the classification framework.

### 3.1. Dataset Preparation

The Herlev Pap Smear dataset, which comprises 917 images, was used and regrouped from seven cytological classes into a binary screening task (normal: 242; abnormal: 675), consistent with clinical triage. This publicly available dataset, introduced by Jantzen et al. [51], is a widely used benchmark for cervical cytology classification. In this study, the dataset was reformulated as a binary classification problem to reflect the primary objective of population-level cervical cancer screening. From the clinical perspective, the key screening decision is to distinguish cases that require further review by a cytopathologist (any abnormal grade) from those that do not (normal), rather than to assign a specific dysplasia grade. The seven-class grading problem is treated as a complementary objective and is discussed as a direction for future work in Section 5.

All images were inspected for readability and resized to a standardized resolution compatible with the ViT architecture while preserving RGB color information. Pixel intensities were normalized using ImageNet statistics to leverage pretrained model weights. Because the Herlev dataset does not include patient-level identifiers, stratified k-fold cross-validation was performed at the image level, with class proportions maintained across folds. The lack of subject-level partitioning is acknowledged as a limitation and discussed further in Section 5. To minimize the risk of data leakage, all preprocessing operations and data augmentations were applied on the fly within the training pipeline.

The Herlev dataset comprises individually segmented single-cell images without patient- or slide-level identifiers. As a result, subject-wise cross-validation could not be performed and image-level stratification was used instead. This limitation may lead to optimistic performance estimates if cells originating from the same patient or slide are present in both the training and testing folds. Because patient-level mappings are unavailable in the Herlev dataset, the extent of such potential data leakage cannot be directly quantified. Although the use of isolated single-cell images reduces the influence of shared whole-slide contextual information, cells from the same patient may still exhibit common staining characteristics, imaging artifacts, or patient-specific morphological characteristics. Consequently, the reported performance should be interpreted as an upper-bound estimate based on internal validation rather than as a measure of patient-level generalization. Future studies should evaluate these models using datasets that include patient identifiers, which enables subject-wise data partitioning and a more realistic assessment of generalizability.

### 3.2. Data Augmentation

Augmentation techniques were designed to increase data diversity while preserving clinically relevant cytological features, which include cell and nuclei morphology as well as chromatin patterns. The augmentation strategies evaluated in this study included color jitter, horizontal flipping, random affine transformation, their pairwise combinations, and the combination of all three, as summarized in Table 1. Horizontal flipping was used to simulate left–right orientation variability. Color jitter introduced modest perturbations in brightness, contrast, and color to reflect staining and image acquisition conditions. Random affine transformation incorporated small rotations, translations, and mild scaling to mimic variability arising from slide-handling and acquisition variability. Thus, rotation, translation, and scaling were treated as components of the random affine transformation rather than as separate augmentation categories. All transformation parameters were restricted to clinically conservative ranges and applied with predefined probabilities to ensure that the augmented images remained biologically plausible. Furthermore, augmentations were applied on the fly during training only. As a result, the nominal dataset size remained unchanged at 917 images, while the effective diversity of training samples increased across epochs.

### 3.3. Class Weighting

Given the class imbalance (normal < abnormal), class-weighted cross-entropy was employed to mitigate bias toward the majority class. For class c with $N_{c}$ samples and a total of N samples across C classes, the class weight $W_{c}$ was defined as follows:

$$W_{c}=\frac{N}{C\times N_{c}}$$

where N is the total number of samples, C is the total number of classes, and $N_{c}$ is the number of samples in class c. For the binary case (C=2), this formulation assigns a larger weight to the minority (normal) class, which thereby penalizes its misclassification more heavily.

The base class weights were derived from the inverse class-frequency formulation. For the binary Herlev task (242 normal, 675 abnormal), this approach assigns a higher base weight to the minority normal class. To further adjust the precision–recall trade-off, five weight-multiplier configurations were evaluated by scaling the base abnormal and normal class weights using factors of 1.0 × 1.0, 0.8 × 0.8, 1.2 × 1.2, 0.7 × 1.3, and 1.3 × 0.7.

The 0.7 × 1.3 configuration was selected empirically, as it achieved the best overall balance, which yielded the highest F1-score and accuracy while maintaining high overall recall. In this setting, the weighted cross-entropy loss penalizes misclassifications in proportion to the assigned class weights.

### 3.4. ViT Model Training

**Figure 3:**
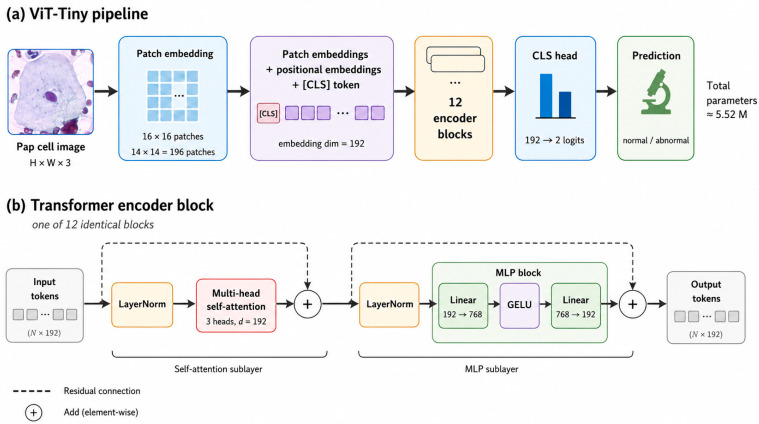
Architecture of the ViT-Tiny model. (**a**) The pipeline splits the input Pap smear cell into 14 × 14 patches, projects them into 192-dimensional tokens with positional embeddings, processes them through 12 identical transformer encoder blocks, and classifies the [CLS] token as normal or abnormal via a linear head. (**b**) Each encoder block consists of LayerNorm, 3 self-attention heads, and an MLP (192 → 768 → 192) with GELU activation, each wrapped in a residual connection. The full model has 5.52 million parameters. Solid arrows indicate the sequential flow of information through the ViT-Tiny pipeline and encoder block.

The primary backbone was ViT-Tiny (vit_tiny_patch16_224; 12 transformer blocks, embedding dimension 192, patch size 16, approximately 5.5 million parameters), a compact Vision Transformer adapted for binary classification by replacing the classification head with a two-logit linear layer. Figure 3 illustrates the overall architecture, which includes the patch-embedding pipeline and the internal structure of a transformer encoder block.

The encoder was initialized with ImageNet pretraining to enhance data efficiency. Training followed a modern optimization recipe, which included AdamW with weight decay, label smoothing, and a learning-rate schedule with warm-up followed by decay. Early stopping and checkpointing were triggered by validation loss to mitigate overfitting.

A full grid search was performed over three key hyperparameters that influence convergence on this dataset, batch size {16, 32, 64}, learning rate {1 × 10^−4^, 5 × 10^−4^, 1 × 10^−3^}, and number of epochs {5, 10, 15}, which resulted in 27 configurations (Section 4.4). The optimizer was AdamW, and all inputs were resized to 224 × 224 RGB images. The primary selection criterion was the mean cross-validation F1-score, with accuracy used as a secondary criterion (Section 3.6).

The optimized ViT-Tiny training configuration was also adopted in a companion benchmarking study [46] to ensure a uniform cross-architecture comparison, which provides additional evidence regarding the computational efficiency and competitive performance of ViT-Tiny.

Formally, the Vision Transformer processes an input image $X\in R^{H\times W\times 3}$ as follows. The image, with height H and width W, is partitioned into N non-overlapping patches of size P×P, where P denotes the patch size:

$$N\;=\;\frac{HW}{P^{2}}$$

Each patch is flattened and linearly projected into a D-dimensional embedding. A learnable class token is prepended to the patch-token sequence, and learnable positional embeddings are added to retain spatial information:

$$z_{0}=\left[x_{class};x_{p}^{1}E;\ldots ;x_{p}^{N}E\right]+E_{pos}$$

where $x_{class}$ denotes the learnable class token, $x_{p}^{i}$ denotes the i-th flattened image patch, E is the linear patch-embedding projection matrix, $E_{pos}$ is the learnable positional embedding, and $z_{0}$ is the initial token sequence passed to the transformer encoder.

Each transformer encoder block consists of layer normalization (LN), multi-head self-attention (MSA), and a multilayer perceptron (MLP), each with residual connections:

$$z_{l}^{\prime }=MSA\left(LN\left(z_{l-1}\right)\right)+z_{l-1}$$

$$z_{l}=MLP\left(LN\left(z_{l}^{\prime }\right)\right)+z_{l}^{\prime }$$

In these equations, l denotes the encoder-layer index. The term $z_{l-1}$ denotes the input token representation to encoder layer l; for the first encoder block, this corresponds to $z_{0}$, the token sequence obtained after adding the patch embeddings, the class token, and positional embeddings. The term $z_{l}^{\prime }$ denotes the intermediate representation after the MSA block and residual connection, and $z_{l}$ denotes the output representation after the MLP block and residual connection.

The self-attention operation is computed as follows:

$$Attention\left(Q,K,V\right)=softmax\left(\frac{QK^{T}}{\sqrt{d_{k}}}\right)V$$

where Q, K, and V are the query, key, and value projections of the input tokens, respectively, and $d_{k}$ denotes the key dimension used to scale the dot-product attention scores.

For ViT-Tiny, the embedding dimension is D=192, with 3 attention heads and an MLP hidden dimension of 768. The model comprises 12 stacked encoder blocks, which result in approximately 5.5 million parameters. For classification, the final-layer class-token representation is passed through a linear head to produce two output logits.

ViT-Tiny was selected over larger transformer variants, such as ViT-Base and ViT-Large, for three main reasons. First, the Herlev dataset contains only 917 images, and higher-capacity transformers are more prone to overfitting in small medical datasets, which typically require large-scale pretraining or substantially larger training sets to fully exploit their capacity. Second, ViT-Tiny has a compact footprint of approximately 5.5 million parameters, which makes it well suited to resource-constrained, often GPU-limited laboratory environments where cervical cancer screening tools are intended to be deployed. Third, the computational efficiency analysis in Section 4.6 demonstrates that ViT-Tiny achieves competitive performance while requiring substantially fewer parameters and less GPU memory than larger baseline models. Taken together, these considerations indicate that ViT-Tiny was chosen not because larger transformers are inherently unsuitable, but because the dataset size, intended deployment context, and empirical efficiency results collectively favor a compact transformer backbone for this task.

### 3.5. Grad-CAM Application

To enhance model interpretability, Grad-CAM was applied to ViT predictions using a transformer-compatible implementation that computes gradients with respect to the final attention-layer feature representations. Because Grad-CAM was originally developed for the spatial feature maps of convolutional neural networks, it was adapted for use with Vision Transformers. Token-level information was projected back to the image space to generate spatial attribution maps.

Grad-CAM was selected as the interpretability method in this study because it produces spatial localization maps that can be directly compared with cytological morphology, which includes nuclei and cytoplasmic regions. This capability makes it well suited for assessing whether the ViT-Tiny model focuses on diagnostically relevant cellular features when making predictions and for examining representative cases of both correct and incorrect classifications.

However, Grad-CAM provides only a qualitative assessment of model behavior. Other interpretability approaches may provide complementary information. In addition, it does not fully capture all transformer-specific attention mechanisms or feature-attribution patterns. Consequently, these visualizations should be interpreted as supportive evidence rather than definitive explanations of the model’s decision-making process.

The sequence of patch tokens output by the final transformer encoder block, excluding the [CLS] token, was reshaped into its original 14 × 14 spatial grid and used as the target activation map in place of a convolutional feature map. Gradients of the target-class logit were computed with respect to these reshaped token activations. These gradients were then averaged across channels to obtain importance weights, which were combined with the corresponding activations and passed through a rectified linear unit (ReLU) operation to generate a class-discriminative localization map. This map was subsequently upsampled to the original input-image resolution.

This reshape-and-attribute procedure represents the standard approach for applying gradient-based localization methods such as Grad-CAM to ViTs. Heatmaps were generated for validation images to visualize the image regions that contributed to the model’s classification decisions. To ensure consistency, visualization parameters, which include smoothing, normalization, and overlay opacity, were held constant across all experimental runs.

This process enables qualitative evaluation of whether the model focuses on clinically relevant morphological features, such as nuclei, irregular cell boundaries, and chromatin distribution patterns. The resulting interpretability findings are presented and discussed in Section 4.

For the representative examples presented in Section 4, a focus score was computed to summarize the spatial concentration of model attention. The Grad-CAM activation map was upsampled to the input-image resolution and normalized to the range [0, 1]. The focus score was defined as the proportion of total Grad-CAM activation obtained within a manually delineated region of interest (ROI), such as a nuclei/abnormal region or a normal-appearing cellular region, relative to the total activation across the entire image:

$$\mathrm{Focus}\;\mathrm{score}\;=\;\frac{\sum_{\mathrm{ROI}}A_{\mathrm{Grad}\text{-}\mathrm{CAM}}}{\sum_{\mathrm{image}}A_{\mathrm{Grad}\text{-}\mathrm{CAM}}}$$

Focus scores range from 0 to 1, with higher values indicating a greater concentration of model attention within the specified ROI. These scores are reported for representative cases to describe attention behavior and are not intended to serve as formal localization-performance metrics.

Fully quantitative localization evaluation, such as pointing-game accuracy or intersection-over-union (IoU) against expert reference masks, requires pixel-level annotations of nuclei or lesion regions. Although the Herlev dataset provides image-level class labels, it does not include segmentation masks or expert-annotated localization ground truth. Consequently, objective localization metrics cannot be computed without the creation of additional manual annotations. The Grad-CAM analysis presented in this study is therefore intended as a qualitative assessment of model interpretability. Future work should incorporate expert-annotated segmentation masks to enable rigorous quantitative evaluation of localization performance.

### 3.6. Evaluation Protocol

For completeness, model performance was evaluated using accuracy, precision, recall, specificity, F1-score, balanced accuracy, Matthews correlation coefficient (MCC), and the area under the receiver operating characteristic curve (ROC-AUC) on the held-out test folds. Because false negatives and false positives have different clinical implications in cervical cancer screening, sensitivity (recall for the abnormal class) and specificity (recall for the normal class) were reported separately. Model discrimination was further assessed using ROC-AUC.

ROC-AUC was computed from the predicted class probabilities to provide a threshold-independent measure of the model’s ability to distinguish between normal and abnormal cells. ROC curves were generated for each replication and averaged across the 10 replications to provide a more robust estimate of discrimination performance. Because the binary Herlev task is class-imbalanced, balanced accuracy and MCC were additionally computed from the pooled cross-validation confusion matrices to provide performance measures that are less sensitive and more robust to class imbalance.

Multiple complementary evaluation metrics are particularly important for imbalanced medical classification problems because reliance on a single metric may provide an incomplete assessment of model performance. The evaluation framework adopted in this study, which includes the cross-architecture comparison presented in Section 4.4, is consistent with the protocol used in the companion benchmarking study [46]. It also aligns with prior health-analytics research on clinical outcome prediction, where comprehensive metric selection has been shown to improve the assessment of both predictive performance and model interpretability [52].

The evaluation metrics are defined as follows:

$$Accuracy=\frac{TP+TN}{TP+TN+FP+FN}$$

$$Precision=\frac{TP}{TP+FP}$$

$$Recall\;(Sensitivity)=\frac{TP}{TP+FN}$$

$$Specificity=\frac{TN}{TN+FP}$$

$$F1-score=\frac{2\cdot (Precision\cdot Recall)}{Precision+Recall}$$

$$Balanced\;Accuracy=\frac{Sensitivity+Specificity}{2}$$

$$MCC=\frac{TP\;\cdot \;TN-FP\;\cdot \;FN}{\sqrt{\left(TP+FP)(TP+FN)(TN+FP)(TN+FN\right)}}$$

where TP, TN, FP, and FN represent true positives, true negatives, false positives, and false negatives, respectively.

To enable consistent comparisons across model configurations, replication-level means and 95% confidence intervals (CIs) were calculated as follows:

$$CI=\bar{x}\pm z\;.\;\frac{s}{\sqrt{n}}\;$$

where x¯ = mean across replications, s = standard deviation across replications, n = number of replications, and z = 1.96 for a 95% confidence interval.

Model interpretability results were summarized using representative Grad-CAM heatmaps. Because the Herlev dataset does not include expert-annotated segmentation marks or localization ground truth, quantitative evaluation of heatmap alignment with clinically relevant regions was not possible. Such analyses can be incorporated in future studies when expert annotations become available.

## 4. Results and Discussion

This section reports experimental outcomes following the same five-stage workflow described in Section 3 and Figure 2: (1) Dataset Preparation, (2) Data Augmentation, (3) Class Weighting, (4) ViT Model Training, and (5) Grad-CAM Interpretability. Organizing results in parallel with methodology enables direct tracing from design choices to performance.

### 4.1. Dataset Preparation Results

The Herlev dataset was successfully preprocessed into 917 images with binary classification (242 normal, 675 abnormal). All images were standardized to RGB format with consistent resolution. ImageNet normalization statistics were applied (mean = [0.485, 0.456, 0.406], std = [0.229, 0.224, 0.225]). Stratified 5-fold cross-validation maintained the 27.8% normal, 72.2% abnormal distribution across all folds.

### 4.2. Data Augmentation Results

Table 1 presents the performance comparison of seven augmentation strategies, three individual augmentation methods and four combined augmentation approaches, evaluated using 5-fold cross-validation with the optimal class-weight configuration.

The effectiveness of the individual augmentation strategies varied considerably. Color Jitter produced the weakest performance, which achieves an accuracy of 89.87% and a precision of 80.40%, which suggests that perturbations in color and staining characteristics may have obscured diagnostically relevant features, such as chromatin texture. Random Affine augmentation achieved a higher accuracy of 91.39% but resulted in lower recall (83.40%), potentially because geometric transformations altered important morphological characteristics, which include nuclei shape and structure. In contrast, Horizontal Flip achieved the best overall performance among the individual augmentation methods, which achieved an accuracy of 94.77% and a recall of 91.30%. These findings suggest that left–right orientation invariance is a beneficial augmentation strategy for cervical cytology images, as it increases data diversity without distorting diagnostically relevant cellular features.

Combined augmentation strategies exhibited limited synergistic effects. Color Jitter + Horizontal Flip achieved an accuracy of 94.33%, slightly below that of Horizontal Flip alone. Color Jitter + Random Affine produced the highest recall (95.00%) but achieved lower precision (84.10%), which indicates an increased rate of false-positive classification. Horizontal Flip + Random Affine achieved an accuracy of 92.26%, while the combination of all three augmentations achieved an accuracy of 94.22%, neither of which provided meaningful improvements over the best-performing individual augmentation strategy.

Overall, the results suggest that augmentation effectiveness depends more on biological plausibility than on the number of augmentation strategies applied. Transformations that preserve diagnostically relevant cellular morphology, such as horizontal flipping, were more effective than those introducing greater visual variability through color or geometric distortions. These findings indicate that simple, clinically plausible augmentation strategies may provide more reliable performance gains than increasingly complex augmentation combinations.

### 4.3. Class Weighting Results

To investigate the effect of class weighting, Table 2 summarizes the systematic evaluation of five class-weight multiplier configurations designed to address class imbalance.

Varying the abnormal-to-normal class weight ratio resulted in distinct precision–recall trade-offs. The optimal configuration was not determined solely by assigning the largest weight to the abnormal class, but by the empirical balance achieved under cross-validation.

Because the Herlev binary task contains fewer normal images than abnormal images, inverse-frequency weighting assigns a higher base weight to the minority normal class. The tested multipliers were therefore used to adjust this baseline and explore different trade-offs between false positives and false negatives.

Case 4 (0.7 × 1.3 multipliers) produced the best overall balance, which achieves the highest F1-score (91.90%) and accuracy (95.64%) while maintaining high overall recall (93.40%). In contrast, the other configurations reduced recall, precision, or the overall F1-score.

Because missed abnormal cells may delay diagnosis, high sensitivity to abnormal cells is particularly important in cervical cancer screening. Class-specific sensitivity and specificity are further examined in the later imbalance-aware performance analysis. The selected weighting scheme provided the best overall balance between recall, precision, and overall classification performance.

### 4.4. ViT Model Training Results

A comprehensive hyperparameter optimization study evaluated 27 combinations of batch sizes (16, 32, 64), learning rates (0.0001, 0.0005, 0.001), and numbers of epochs (5, 10, 15). All configurations were assessed using stratified 5-fold cross-validation. Table 3 reports the average performance across folds for all 27 configurations, with the top-performing settings highlighted in bold.

A learning rate of 0.0001 consistently outperformed higher values, which ensured stable convergence and prevented accuracy loss caused by overshooting the loss minimum during optimization. Batch size influenced training efficiency: batch size 16 required more epochs due to noisier gradient estimates, whereas batch size 32 achieved the best overall balance and the highest accuracy (96.51%). In contrast, batch size 64 showed a slight performance decline, likely due to reduced gradient diversity. Training duration analysis indicated that 15 epochs provided optimal convergence, while shorter training runs appeared to result in underfitting.

To evaluate the stability of the selected configurations, each was replicated 10 times using different random initializations. This replication reduces the variability introduced by random weight initialization and data shuffling, which results in a more reliable estimate of model performance. In Table 4, “CV” denotes cross-validation performance computed on the held-out fold for each split, while “App” denotes application-level evaluation on the full dataset used to train the final model. Table 4 reports the mean and 95% confidence interval across these replications for both cross-validation and application-level performance.

As shown in Table 4, B32_E15 (batch size of 32 and 15 epochs) was selected as the best configuration because it achieved the highest cross-validation F1-score (90.63%), while maintaining competitive cross-validation accuracy (94.89%) with a narrow 95% confidence interval, which indicates stable performance across replications. Although B64_E15 achieved a slightly higher cross-validation accuracy (95.01%), B32_E15 provided a better overall balance between precision and recall. F1-score was used as the primary selection metric, with accuracy treated as a secondary metric.

The application-level results are included in Table 4 for completeness because they were obtained by resubstitution on the full dataset used to train the final model and may therefore overestimate generalization performance. Consequently, all comparisons in this study are based exclusively on cross-validation results. The relatively wider confidence intervals observed for some application-level metrics further indicate that these estimates are not a reliable basis for model selection.

**Figure 4:**
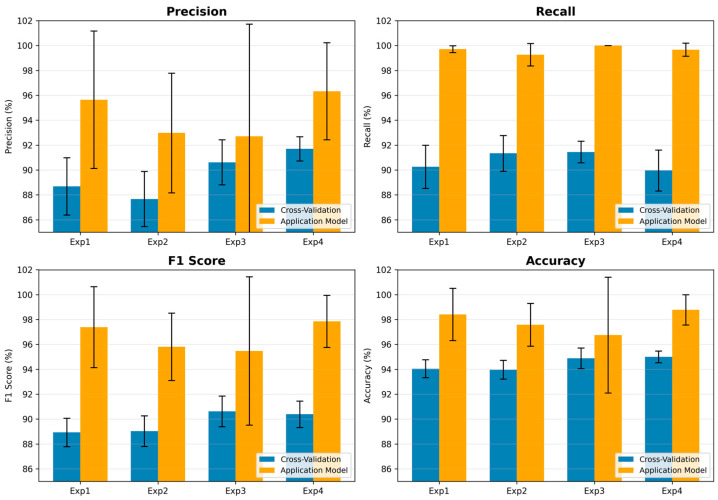
Performance comparison across the four ViT-Tiny configurations.

Figure 4 summarizes the variation in performance across the evaluated configurations. Formal pairwise statistical testing was then conducted to assess whether observed differences in accuracy and F1-score were statistically significant. Pairwise comparisons were reported for accuracy and F1-score because, in this binary screening setting, F1-score provides a balanced measure by integrating both precision and recall.

For each pairwise comparison, let $d_{i}$ denote the performance difference between two configurations at replication i. The mean difference $\bar{d}$ and sample variance $s_{d}^{2}$ were computed over n=10 replications. Because repeated cross-validation runs share overlapping training folds and are therefore not independent, the corrected resampled t-statistic of Nadeau and Bengio [53] was used:

$$t_{NB}=\frac{\bar{d}}{\sqrt{\left(\frac{1}{n}+\frac{n_{test}}{n_{train}}\right)s_{d}^{2}}}$$

In the 5-fold cross-validation setting, the ratio of test to training samples is given by:

$$\frac{n_{test}}{n_{train}}=\frac{\left(\frac{1}{5}\right)}{\left(\frac{4}{5}\right)}=0.25$$

The Holm–Bonferroni procedure was then applied across the six pairwise comparisons to control the family-wise error rate.

The results of these pairwise comparisons are reported in Table 5 and Table 6 for accuracy and F1-score, respectively. After applying the Nadeau–Bengio corrected resampled t-test together with the Holm–Bonferroni adjustment across the six pairwise comparisons, none of the differences remained statistically significant (all adjusted p>0.05). This indicates that the four evaluated ViT-Tiny configurations are statistically comparable in terms of classification performance.

**Figure 5:**
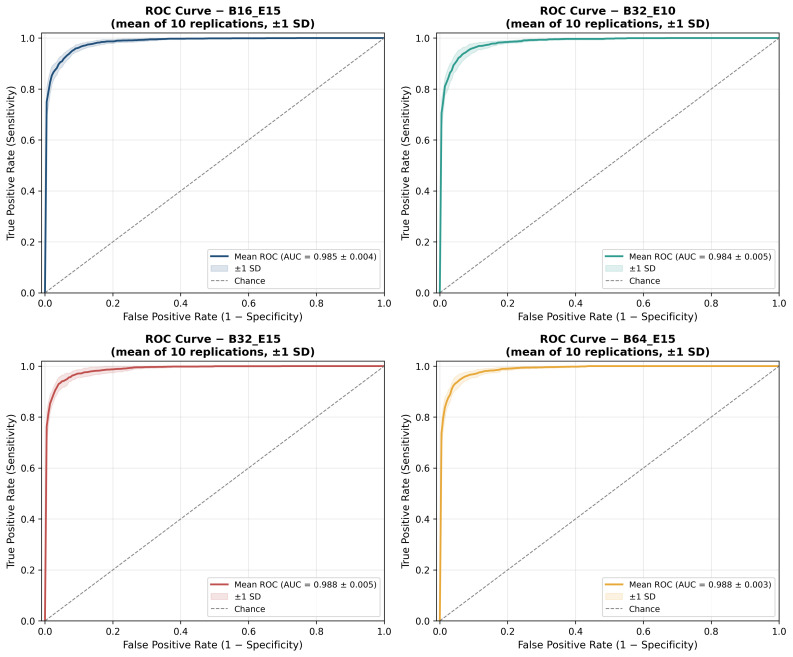
ROC curves for the four evaluated ViT-Tiny configurations on the Herlev binary task (mean across 10 replications; shaded bands indicate ±1 standard deviation). Mean ROC-AUC values were 0.985 ± 0.004 (B16_E15), 0.984 ± 0.005 (B32_E10), 0.988 ± 0.005 (B32_E15), and 0.988 ± 0.003 (B64_E15), which indicate strong and consistent discrimination performance across all configurations.† Macro-F1 score calculated from the class-specific F1-scores reported in the original study.

Figure 5 presents the ROC curves for all four configurations, each computed as the mean across ten replications with shaded ±1 standard deviation bands. All configurations demonstrate high and closely aligned discrimination performance, with mean ROC-AUC values ranging from 0.984 to 0.988 and low standard deviations (≤0.005), consistent with the statistical equivalence indicated by the corrected pairwise tests.

**Figure 6:**
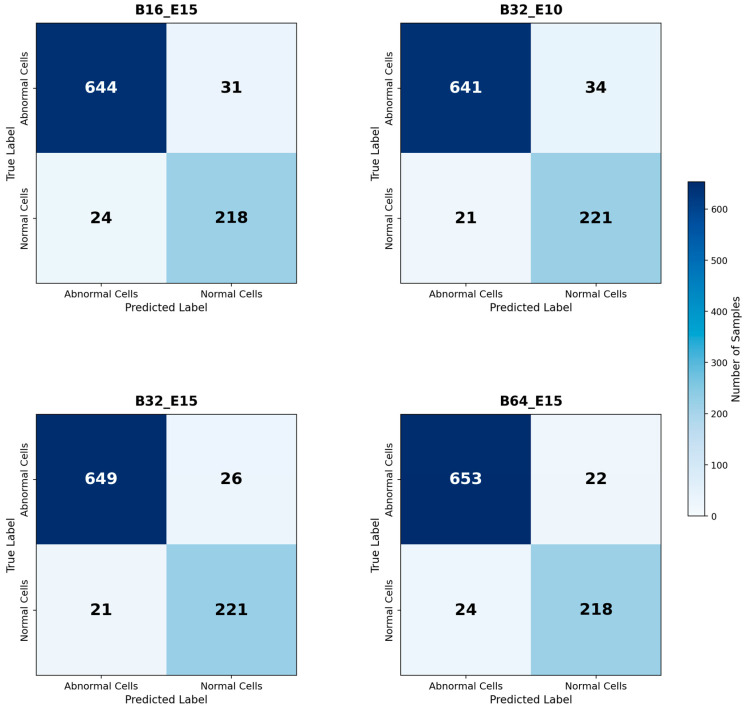
Binary confusion matrices for the main cross-validation experimental settings, averaged over 10 replications. Rows represent true labels and columns represent predicted labels.

To further clarify the screening-level error patterns, Figure 6 presents binary confusion matrices for the main cross-validation experimental settings, averaged over 10 replications. The matrices show strong discrimination between abnormal and normal cells, with most abnormal cells correctly classified and relatively few false negatives across all evaluated configurations.

To complement the confusion-matrix analysis and account for class imbalance, Table 7 reports sensitivity, specificity, balanced accuracy, and MCC for the four ViT-Tiny configurations. Across configurations, balanced accuracy ranged from 92.75% to 93.74%, while MCC ranged from 0.847 to 0.871, which indicate consistently strong binary classification performance under the class-imbalanced Herlev dataset setting.

Sensitivity represents recall of the abnormal class, while specificity represents recall of the normal class. Balanced accuracy is computed as the average of sensitivity and specificity and MCC denotes Matthews correlation coefficient. All metrics were calculated from pooled binary cross-validation confusion matrices averaged over 10 replications.

### 4.5. Grad-CAM Interpretability Results

Gradient-weighted Class Activation Mapping was applied to the best-performing configuration (B32_E15) to visualize model decision-making patterns and provide transparency in the classification process.

**Figure 7:**
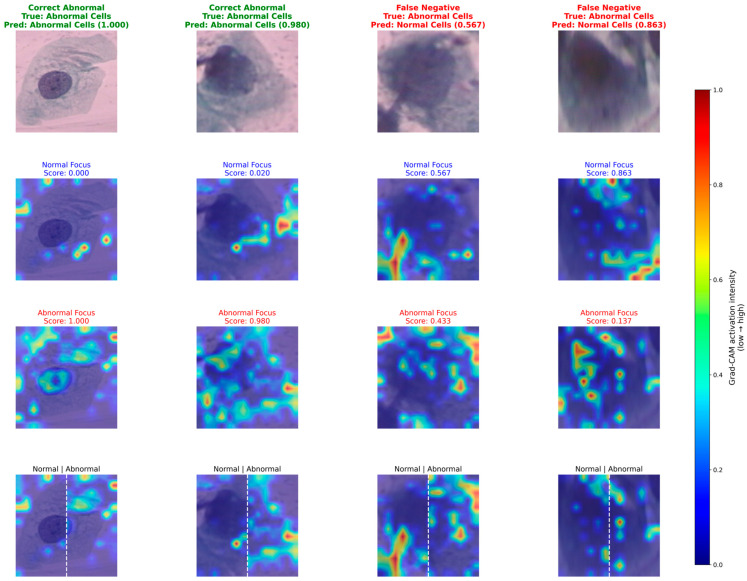
Grad-CAM analysis of correct abnormal classifications and false negative cases.

Figure 7 presents Grad-CAM visualizations for correctly classified abnormal cells and false-negative cases. The reported “focus scores” denote the normalized proportion of total Grad-CAM activation that falls within manually delineated regions of interest (nuclei/abnormal regions versus normal-appearing regions), scaled to the range [0, 1]. These values are provided for illustrative representative cases rather than as a dataset-wide quantitative metric.

For the correctly classified abnormal cells, the model exhibited strong localization on abnormal/nuclei regions, with abnormal focus scores ranging from 0.980 to 1.000 and correspondingly low normal-region focus scores ranging from 0.000 to 0.020. In contrast, false-negative cases showed higher normal-region focus scores (0.567–0.863) and lower abnormal-region focus scores (0.137–0.433), which indicate that the model placed greater emphasis on normal-appearing regions rather than diagnostically relevant abnormal features in these samples.

Quantitative localization evaluation (e.g., pointing-game accuracy or IoU against expert annotations) requires pixel-level segmentation masks, which are not available in the Herlev dataset. The focus scores reported here should be interpreted as descriptive summaries of Grad-CAM activation for representative cases rather than as validated, dataset-wide localization metrics. Future work should incorporate expert-annotated segmentation data to enable rigorous quantitative assessment of localization performance.

The misclassified cases suggest several possible sources of error. False-positive predictions may occur when staining artifacts, cell overlap, or benign atypical regions produce texture patterns that resemble abnormal chromatin, which lead the model to focus on non-diagnostic regions. False-negative predictions, in contrast, may occur when abnormal nuclei are partially obscured by overlapping cells or debris, or when cytological features are subtle or ambiguous, which cause the model’s attention to shift toward normal-appearing regions rather than the abnormal nuclei regions.

These observations suggest several directions for future work, which include cell-overlap-aware preprocessing or segmentation, stain normalization, artifact-robust augmentation, and uncertainty-aware prediction to flag ambiguous cases for cytopathologist review.

**Figure 8:**
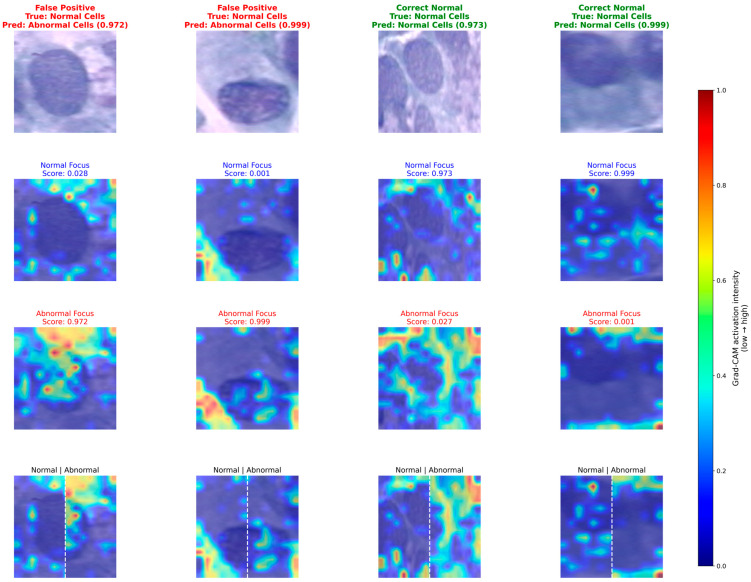
Grad-CAM analysis of correct normal classifications and false positive cases.

Figure 8 illustrates the attention patterns for false-positive errors and correctly classified normal cells. In the false-positive cases, the model exhibits high abnormal-region focus scores (0.972–0.999) and very low normal-region focus scores (0.001–0.028), which indicate strong attention to regions interpreted as abnormal despite the true label being normal. These patterns may reflect benign morphological or staining features that resemble abnormal cytological characteristics.

In contrast, correctly classified normal cells show high normal-region focus scores (0.973–0.999) with minimal abnormal-region focus scores (0.001–0.027), which suggest that the model appropriately concentrates its attention on normal-appearing regions in these representative cases.

The attention patterns shown in Figure 7 and Figure 8 are generally consistent with established cytopathological criteria in these representative examples, which include nuclei morphology, chromatin distribution, and cellular boundaries, the same features used by cytopathologists for diagnosis. This correspondence suggests that the Vision Transformer model can learn clinically relevant visual representations for cervical cancer screening. However, further quantitative validation using expert annotations is required to confirm the robustness and clinical reliability of these findings.

### 4.6. Comparison with CNN Baselines and Computational Efficiency

To place the performance of ViT-Tiny in the context of established convolutional architectures, a controlled cross-architecture benchmark was conducted in the authors’ companion study [46]. In that study, ViT-Tiny and four widely used CNN baselines (ResNet50, EfficientNet-B0, VGG16, and DenseNet121) were evaluated on the same Herlev binary classification task using a uniform training protocol, matched data splits, and identical replication procedures.

Under these controlled conditions, ViT-Tiny achieved classification performance comparable to the strongest CNN baselines (VGG16 and DenseNet121), with paired statistical testing indicating no significant differences between them. At the same time, ViT-Tiny significantly outperformed ResNet50 and EfficientNet-B0 [46]. These findings suggest that, among competitive modern architectures, the choice of backbone is not the primary determinant of classification accuracy on this dataset, which shifts the practical focus toward efficiency and deployment considerations. Detailed cross-architecture results are reported in the companion study [46]. The results presented in the current study, including the 94.89% cross-validation accuracy of the optimal B32_E15 configuration (Section 4.4), were obtained under a different optimization protocol and therefore constitute the primary findings of this work.

Beyond predictive performance, the companion study also evaluated computational efficiency on an NVIDIA A100 GPU. Once classification performance reaches a comparable level across architectures, efficiency becomes a key consideration for real-world deployment.

As shown in Table 8, ViT-Tiny requires approximately 24 times fewer parameters than VGG16 (5.52 M vs. 134.27 M), approximately 15 times less peak GPU memory (112.7 MB vs. 1725.0 MB), and achieves roughly 2.3 times higher batch-level throughput (4089 vs. 1808 images/s). Although VGG16 exhibits the lowest single-image latency, its substantially larger parameter count and memory footprint limit overall batch-level throughput. Compared with the more compact DenseNet121, ViT-Tiny uses slightly fewer parameters, substantially less peak GPU memory, and achieves approximately 2.1 times higher throughput. For large-scale screening applications, batch-level throughput and memory efficiency are more relevant deployment metrics than single-image latency, which further support the selection of ViT-Tiny as a lightweight and deployment-oriented architecture.

These efficiency advantages are particularly important in resource-constrained environments, where access to high-performance computing infrastructure may be limited. The combination of competitive classification performance and substantially lower computational requirements provides a practical justification for selecting a compact Vision Transformer architecture over larger CNN backbones for cervical cancer screening applications. Detailed latency, throughput, and memory measurements are reported in [46].

### 4.7. Comparison with Prior Work

To position the present results within the context of prior work on the same dataset, Table 9 compares binary cervical cytology classification studies using the Herlev Pap smear dataset in terms of methodology, reported performance, validation strategy, data-splitting approach, and methodological rigor. Early studies primarily relied on classical machine learning proposed by Marinakis et al. [54]. More recent research has increasingly adopted deep learning architectures and, most recently, transformer-based models.

Among the studies included in the comparison, the proposed ViT-Tiny framework is the only one that simultaneously incorporates stratified cross-validation, statistical significance testing, and model interpretability analysis. Although several earlier studies reported higher headline accuracy values, those results were typically obtained using a single train/test split without cross-validation or significance testing. Such evaluation protocols are generally associated with greater variance and a higher risk of optimistic performance estimates, which make direct comparisons with cross-validated results difficult.

Recent transformer-based and deep-learning studies further contextualize the present work. A transfer-learning approach combining VGG19 and ResNet50 feature extraction with a Naive Bayes classifier was evaluated on the Herlev dataset [48], while a Vision Transformer with shifted patch tokenization was applied to the MobileODT colposcopy dataset and achieved 91% accuracy using a single 70/30 train/test split [47]. A Swin Transformer combined with a deep-learning ensemble has also been proposed for colposcopy screening [49], and a hybrid Vision Transformer ensemble incorporating DenseNet201, Xception, and InceptionResNetV2 was evaluated on the Mendeley LBC and SIPaKMeD datasets [50].

With the exception of the Herlev component in [48], these studies were conducted on datasets and classification tasks that differ substantially from the binary single-cell Herlev problem considered here, which include multi-class colposcopy and liquid-based cytology datasets. Consequently, direct numerical comparisons should be interpreted with caution. Rather than focusing solely on predictive performance, the present study emphasizes reproducible model optimization, stratified cross-validation with replication, corrected statistical significance testing, and Grad-CAM interpretability using a compact, computationally efficient, and deployment-oriented Vision Transformer framework.

As Table 9 indicates, the proposed ViT-Tiny model achieves competitive cross-validated accuracy (94.89%) while incorporating stratified cross-validation, pairwise statistical testing, and Grad-CAM-based interpretability aligned with established cytopathological criteria. Among the cross-validated studies, the ResNet-101 approach with integrated gradients [58] represents the most comparable benchmark, which reports a binary classification accuracy of 94.0% on the Herlev dataset using 4-fold cross-validation. The present approach exceeds that performance by approximately 0.9 percentage points while employing replicated stratified 5-fold cross-validation and corrected pairwise statistical testing.

Another study using 5-fold cross-validation reported a higher accuracy of 99.5% [56]. However, that work did not include statistical significance testing or clinical interpretability analysis, which limits direct comparison from a methodological-rigor perspective. The remaining studies primarily relied on single train/test splits, which generally provide less robust estimates of generalization performance and are more susceptible to optimistic performance bias than replicated cross-validation protocols.

## 5. Conclusions, Limitations, and Future Work

This study demonstrates that Vision Transformers can serve as accurate and interpretable tools for automated cervical cancer screening. By restructuring Pap smear classification into a binary screening task, the evaluation was aligned with clinical practice, where the primary objective is to distinguish cells requiring further review from those that do not. The results show that an optimized ViT-Tiny model can achieve approximately 95% cross-validation accuracy. Furthermore, Nadeau–Bengio corrected pairwise tests with Holm–Bonferroni adjustment indicated that the top-performing configurations were statistically comparable. Interpretability analysis suggested that, in the representative cases examined, model attention corresponded to clinically meaningful morphological features, which supports the potential utility of Vision Transformers as decision-support tools pending broader validation. Accordingly, the proposed system should be regarded as a research-stage screening-support model rather than a clinically deployable tool, and the reported results should not be interpreted as evidence of readiness for clinical deployment.

Several limitations should be acknowledged. First, the evaluation was conducted exclusively on the Herlev dataset, which contains only 917 images and may not fully capture the variability encountered across clinical settings. Differences in staining protocols, imaging devices, patient demographics, HPV prevalence, lesion-grade distribution, cell sampling procedures, and slide preparation practices may affect model generalizability. In addition, because the Herlev dataset does not provide patient- or slide-level identifiers, patient-wise cross-validation could not be performed [60]. Consequently, the reported performance should be interpreted as an internal validation estimate rather than evidence of patient-level or clinical generalization. Second, although the binary classification formulation is clinically relevant for screening applications, it simplifies the original seven-class Herlev taxonomy into a normal-versus-abnormal decision. This design was intentionally chosen to reflect the primary purpose of screening: identifying potentially abnormal cells for further expert review. However, it does not assess the model’s ability to distinguish among the finer cytological subtypes represented in the original multi-class grading scheme. The present findings should be interpreted as evidence of screening-level discrimination rather than comprehensive diagnostic categorization. Future work should extend the framework to the original seven-class problem and report class-specific performance measures, which include per-class F1-scores and multi-class confusion matrices. Third, the study relied on existing dataset annotations and did not evaluate inter-rater reliability, which may introduce label noise. Fourth, the Grad-CAM analysis provides qualitative interpretability for representative cases but does not constitute expert-validated quantitative localization. In addition, Grad-CAM represents only one visualization-based explanation technique and may not fully capture transformer-specific attention mechanisms or feature-attribution behavior. Future studies should incorporate complementary interpretability approaches, such as attention rollout, transformer attention maps, SHAP, and integrated gradients, together with expert-annotated localization assessments to provide a more comprehensive evaluation of model decision-making. Finally, the augmentation strategy, class-weight configuration, and hyperparameter settings were selected using cross-validation results from the same Herlev dataset. As a result, some degree of model-selection bias may remain. The reported performance should therefore be interpreted as an internally optimized estimate rather than an unbiased estimate of external generalization.

Future work should validate the proposed framework on independent datasets such as SIPaKMeD and, ideally, larger multi-center cohorts that include patient-level identifiers to enable subject-wise data partitioning. Nested cross-validation, independent hold-out test sets, and cross-dataset transfer experiments should be employed to separate model selection from final performance estimation. Additional work should also incorporate expert-consensus labeling, quantitative localization based on pixel-level annotations, and prospective evaluation within real-world screening workflows that involve cytotechnologists and pathologists. Such studies should assess not only diagnostic performance, but also workflow integration, operational efficiency, user acceptance, and the practical value of AI-assisted cytopathology in routine clinical practice. In particular, the model’s diagnostic performance should be directly benchmarked against that of expert cytopathologists to establish its standalone and assistive value before any clinical-use claims can be made.

## Figures and Tables

**Table 1:** Augmentation techniques evaluation (5-fold cross-validation).

Augmentation Strategy	Precision (%)	Recall (%)	F1-Score	Accuracy (%)
Color Jitter	80.40	90.60	83.30	89.87
Horizontal Flip	**89.70**	**91.30**	**90.00**	**94.77**
Random Affine	86.90	83.40	83.10	91.39
Color Jitter + Horizontal Flip	89.10	90.10	89.40	94.33
Color Jitter + Random Affine	84.10	95.00	88.70	93.23
Horizontal Flip + Random Affine	83.60	89.70	85.70	92.26
All Three Combined	89.40	89.70	89.10	94.22

Bold values indicate the best-performing result for each metric.

**Table 2:** Class weight optimization results (5-fold cross-validation).

Case No.	Weight Multiplier	Abnormal Weight	Normal Weight	Precision (%)	Recall (%)	F1-Score (%)	Accuracy (%)
1	1.0 × 1.0	0.68	1.90	92.10	84.30	87.40	93.67
2	0.8 × 0.8	0.54	1.52	84.40	90.60	85.80	91.93
3	1.2 × 1.2	0.82	2.27	83.00	93.00	86.70	91.72
4	0.7 × 1.3	0.48	2.46	90.90	93.40	91.90	95.64
5	1.3 × 0.7	0.88	1.33	90.70	88.80	89.70	94.55

**Table 3:** Full hyperparameter optimization results (27 configurations). Bold rows indicate the top-performing configurations selected for further analysis.

Experiment No.	Batch Size	Learning Rate	Epochs	Precision (%)	Recall (%)	F1-Score (%)	Accuracy (%)
1	16	0.0001	5	79.14	90.93	84.28	90.95
2	16	0.0001	10	89.91	90.07	89.66	94.55
**3**	**16**	**0.0001**	**15**	**93.36**	**90.52**	**91.82**	**95.75**
4	16	0.0005	5	53.84	87.17	61.00	65.32
5	16	0.0005	10	58.96	84.23	68.55	78.96
6	16	0.0005	15	65.70	83.97	72.31	82.54
7	16	0.001	5	53.53	79.31	58.01	62.83
8	16	0.001	10	53.03	83.38	60.91	68.72
9	16	0.001	15	59.24	78.87	63.69	73.07
10	32	0.0001	5	85.31	90.47	87.56	93.34
**11**	**32**	**0.0001**	**10**	**90.47**	**95.06**	**92.59**	**95.96**
**12**	**32**	**0.0001**	**15**	**96.23**	**90.49**	**93.10**	**96.51**
13	32	0.0005	5	56.57	77.67	61.21	70.13
14	32	0.0005	10	68.24	78.98	71.36	82.43
15	32	0.0005	15	53.52	95.88	68.11	74.94
16	32	0.001	5	67.09	60.26	57.91	77.53
17	32	0.001	10	52.15	81.38	61.44	70.77
18	32	0.001	15	49.43	86.44	60.69	67.26
19	64	0.0001	5	78.96	95.46	86.05	91.71
20	64	0.0001	10	84.55	95.03	89.39	94.00
**21**	**64**	**0.0001**	**15**	**93.83**	**92.15**	**92.87**	**96.29**
22	64	0.0005	5	50.99	84.75	59.55	67.39
23	64	0.0005	10	61.60	93.38	73.71	82.02
24	64	0.0005	15	71.84	82.19	75.72	85.72
25	64	0.001	5	51.95	69.81	57.18	72.73
26	64	0.001	10	42.27	88.89	55.77	61.49
27	64	0.001	15	58.11	88.87	69.27	78.17

**Table 4:** Comprehensive experimental results (10 replications), reported as mean [95% CI].

Configuration (B = Batch Size, E = Epochs)	CV Precision (%)	CV Recall (%)	CV F1-Score (%)	CV Accuracy (%)	App Precision (%)	App Recall (%)	App F1-Score (%)	App Accuracy (%)
B16_E15	88.69 [86.27, 91.12]	90.26 [88.44, 92.08]	88.93 [87.73, 90.14]	94.05 [93.28, 94.81]	95.65 [89.83, 100.00]	99.71 [99.41, 100.00]	97.39 [93.96, 100.00]	98.41 [96.19, 100.00]
B32_E10	87.67 [85.34, 89.99]	91.34 [89.83, 92.85]	89.03 [87.73, 90.32]	93.97 [93.18, 94.76]	92.98 [87.91, 98.05]	99.26 [98.31, 100.00]	95.81 [92.95, 98.67]	97.58 [95.76, 99.40]
B32_E15	90.62 [88.71, 92.54]	91.45 [90.54, 92.36]	90.63 [89.33, 91.92]	94.89 [94.01, 95.77]	92.70 [83.18, 100.00]	100.00 [100.00, 100.00]	95.48 [89.21, 100.00]	96.75 [91.85, 100.00]
B64_E15	91.70 [90.68, 92.72]	89.96 [88.22, 91.70]	90.39 [89.28, 91.51]	95.01 [94.51, 95.50]	96.33 [92.23, 100.00]	99.67 [99.11, 100.00]	97.86 [95.66, 100.00]	98.78 [97.50, 100.00]

**Table 5:** Pairwise comparison results for accuracy (Nadeau–Bengio corrected resampled *t*-test with Holm–Bonferroni adjustment).

Comparison	Mean_1_ (Config A)	Mean_2_ (Config B)	Diff (Config A − Config B)	*p* (NB + Holm)	*p* (Nadeau–Bengio)	Significant? (*p* < 0.05)
B16_E15 vs. B32_E10	94.05	93.97	+0.076	1.000	0.944	No
B16_E15 vs. B32_E15	94.05	94.89	−0.839	1.000	0.334	No
B16_E15 vs. B64_E15	94.05	95.01	−0.960	1.000	0.203	No
B32_E10 vs. B32_E15	93.97	94.89	−0.915	1.000	0.507	No
B32_E10 vs. B64_E15	93.97	95.01	−1.035	0.950	0.158	No
B32_E15 vs. B64_E15	94.89	95.01	−0.121	1.000	0.898	No

**Table 6:** Pairwise comparison results for F1-score (Nadeau–Bengio corrected resampled *t*-test with Holm–Bonferroni adjustment).

Comparison	Mean_1_ (Config A)	Mean_2_ (Config B)	Diff (Config A − Config B)	*p* (NB + Holm)	*p* (Nadeau–Bengio)	Significant? (*p* < 0.05)
B16_E15 vs. B32_E10	88.93	89.03	−0.094	1.000	0.958	No
B16_E15 vs. B32_E15	88.93	90.63	−1.691	1.000	0.226	No
B16_E15 vs. B64_E15	88.93	90.39	−1.457	1.000	0.316	No
B32_E10 vs. B32_E15	89.03	90.63	−1.597	1.000	0.442	No
B32_E10 vs. B64_E15	89.03	90.39	−1.363	1.000	0.331	No
B32_E15 vs. B64_E15	90.63	90.39	+0.235	1.000	0.881	No

**Table 7:** Imbalance-aware performance metrics for the four ViT-Tiny configurations on the Herlev binary cross-validation task.

Configuration	Sensitivity (%)	Specificity (%)	Balanced Accuracy (%)	MCC
B16_E15	95.41	90.08	92.75	0.847
B32_E10	94.96	91.32	93.14	0.849
B32_E15	96.15	91.32	93.74	0.869
B64_E15	96.75	90.08	93.41	0.871

**Table 8:** Computational efficiency measured on an NVIDIA A100 GPU. Latency was averaged over 100 forward passes following a 20-pass warm-up, and throughput was measured using a batch size of 32.

Model	Params (M)	Single-Image Latency (ms)	Throughput (img/s)	Peak GPU (MB)
ViT-Tiny	5.52	5.20	4089	112.7
ResNet50	23.51	6.53	2772	445.2
EfficientNet-B0	4.01	8.38	3642	423.5
VGG16	134.27	2.18	1808	1725.0
DenseNet121	6.96	16.96	1904	369.4

**Table 9:** Comparison of binary cervical cytology classification studies using the Herlev dataset. All studies were evaluated on the Herlev dataset (917 images) for binary classification of normal versus abnormal cells. Methodological-rigor criteria are reported as Yes/No.

Study	Method	Acc (%)	F1 (%)	Validation Technique	Split Type	Dataset	Statistical Test	Interpretability
Yilmaz et al. [55]	XGBoost/k-NN and Custom CNN	85.0/93.0	87.0/95.0	Train/test split (85/15)	Single split	Herlev (917)	No	No
Ghoneim et al. [56]	CNN + extreme learning machine	99.5	—	5-fold CV	Cross-validation	Herlev (917)	No	No
Deo et al., CerviFormer [57]	Cross-attention transformer	94.57	92.5 †	Train/test split (90/10)	Single split	Herlev (917)	No	No
Pirovano et al. [58]	ResNet-101 + Integrated Gradients	94.0	96.0	4-fold CV	Cross-validation	Herlev (917)	No	Yes (Integrated Gradients)
Kaur et al. [59]	ResNet50 (best of 16 TL models)	95.0	94.0	Train/val/test (60/20/20)	Single split	Herlev (917)	No	No
Sharma et al. [48]	VGG19 + ResNet50 feature extraction with Naive Bayes	91.15	90.5	Train/test split	Single split	Herlev (917)	No	No
**Proposed Methodology**	**ViT-Tiny (~5.5 M) + Grad-CAM**	**94.89**	**90.63**	**Stratified 5-fold CV (×10 reps)**	**Cross-validation**	**Herlev (917)**	**Yes (Nadeau–Bengio + Holm)**	**Yes (Grad-CAM)**

† Macro-F1 score calculated from the class-specific F1-scores reported in the original study.

## Data Availability

The Herlev Pap smear dataset used in this study is publicly available from its original source.
